# Analysis of risk factors for perioperative hyperamylasemia in kidney transplant recipients

**DOI:** 10.1080/0886022X.2025.2529444

**Published:** 2025-07-23

**Authors:** Yang Zhang, Liubing Xia, You Luo, Jinhua Zhang, Zoufu Tang, Xiaorong Chen, Ning Na

**Affiliations:** a Department of Kidney Transplantation, The Third Affiliated Hospital of Sun Yat-sen University, Guangzhou, China; b Organ Transplantation Research Institute, The Third Affiliated Hospital of Sun Yat-sen University, Guangzhou, Guangdong, China

**Keywords:** Renal transplantation, perioperative hyperamylasemia, hyperamylasemia, phosphate, calcineurin inhibitors

## Abstract

**Background:**

Perioperative hyperamylasemia has been observed in several kidney transplant recipients (KTRs) at our center. However, there are currently no published reports on this observation. This study aimed to identify the risk factors associated with perioperative hyperamylasemia in KTRs.

**Methods:**

The data from 540 deceased-donor kidney recipients in our hospital from January 2020 to December 2023 were retrospectively analyzed. Variables such as gender, past medical history, relevant laboratory tests, and trough concentration of calcineurin inhibitors (CNIs) at the time of serum amylase maximum were collected for all patients. Univariate and multivariate logistic regression analyses were used to determine the risk factors associated with perioperative hyperamylasemia.

**Results:**

Among all KTRs, 153 patients (28.3%) developed perioperative hyperamylasemia. Multivariate logistic regression analysis indicated that preoperative serum phosphate (odds ratio [OR] = 1.62, 95% confidence interval [CI]: 1.160 − 2.266, *p* = 0.005), preoperative serum amylase (OR = 1.01, 95% CI: 1.006 − 1.015, *p* < 0.001), and high perioperative CNIs trough concentration (OR = 2.335, 95% CI: 1.560 − 3.494, *p* < 0.001) were risk factors associated with perioperative hyperamylasemia. In addition, we used all three as a hybrid model to predict perioperative hyperamylasemia, which demonstrated good predictive value (area under the ROC curve [AUC] = 0.687, 95% CI: 0.64–0.734).

**Conclusion:**

Elevated preoperative serum phosphate levels, preoperative serum amylase levels, and high perioperative CNIs trough concentrations are risk factors for perioperative hyperamylasemia. This study may provide valuable insights for clinicians to identify the causes of perioperative hyperamylasemia and formulate prevention and treatment strategies.

## Introduction

Acute pancreatitis (AP) is a common inflammatory disease of the exocrine pancreas that causes severe abdominal pain and multiple organ dysfunction that may lead to pancreatic necrosis and persistent organ failure, with a mortality of 1–5% [1]. AP is an infrequent yet severe complication after kidney transplantation. The incidence of AP after kidney transplantation ranges from 1.2 to 6.8% [2–4]. Hyperamylasemia is an early sign of pancreatic cell damage and one of the diagnostic criteria for pancreatitis. In AP, serum amylase levels in the blood rapidly rise to at least three times the upper limit of normal, which is defined as hyperamylasemia [5]. Moreover, in the presence of ongoing immunosuppressants, atypical presentations are not uncommon. Other causes of hyperamylasemia include pancreas-related diseases (pancreatic tumors, pancreatic duct blockage), malignancies (breast, colon, lung, and ovarian cancers), salivary gland and fallopian tube disorders, intestinal ischemia, perforated peptic ulcer, and chronic renal insufficiency [6–8].

The presence of hyperamylasemia in the perioperative period is often a cause for concern for clinicians, alerting them to the possibility of pancreatitis and treating it symptomatically.

Perioperative hyperamylasemia has been observed in some kidney transplant recipients (KTRs) at our institution. Our study’s cohort is based on 4 years of data from a tertiary care hospital. This single-center retrospective study was designed to analyze the risk factors associated with perioperative hyperamylasemia in KTRs.

## Materials and methods

### Study design and participants

**Figure 1.**
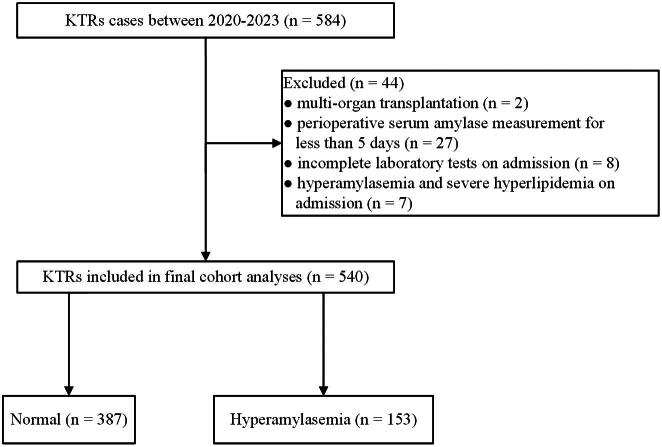
Study design and cohort build-up.

Deceased-donor kidney recipients at the Third Affiliated Hospital of Sun Yat-sen University, Guangzhou, China, between January 2020 and December 2023 were considered for inclusion in this retrospective study. Exclusion criteria included multi-organ transplantation, perioperative serum amylase measurement for less than 5 days, incomplete laboratory tests on admission, as well as hyperamylasemia and hyperlipidemia on admission. Serum amylase is elevated within 6–12 h after an episode of AP. Amylase has a short half-life of about 10 h; in the absence of complications, it returns to normal within 3–5 days. Therefore, in this study, patients with perioperative amylase measurements of less than 5 days were excluded. And Amylase levels in our hospital’s clinical laboratory are measured using the rate method. Figure 1 illustrates the detailed process. Demographic data for the recipients and donors were collected from the China organ transplant response system (COTRS) and the Hospital Information System (HIS), respectively. COTRS is the only official registry platform designated by the National Health Commission of China for solid organ donation from deceased citizen donors, matching, and allocation (https://www.cot.org.cn/). This study was approved by the Research Ethics Committee of the Third Affiliated Hospital of Sun Yat-sen University (SL-II2024-224-01). This research abides by the Declaration of Helsinki. The clinical and research activities being reported are consistent with the Principles of the Declaration of Istanbul outlined in the ‘Declaration of Istanbul on Organ Trafficking and Transplant Tourism’.

### Study variables

The patient’s body mass index (BMI), biologic sex, age, and past medical history (hepatitis, chronic smoking, chronic drinking, hypertension, diabetes, cerebral infarction, coronary heart disease (CAD), polycystic kidney disease (PKD), pancreatitis, dialysis types, Induction types, etc.) were all retrieved by HIS. Preoperative laboratory measurements included aspartate aminotransferase (AST), alanine aminotransferase (ALT), albumin (ALB), total cholesterol (TC), triglycerides (TG), low-density lipoprotein Cholesterol (LDL-C), serum calcium, serum phosphorus, and serum amylase. Postoperative variables included immune induction regimen, type of CNIs, serum amylase levels and the type and trough concentration of CNIs at serum amylase maximum. Plasma calcium corrected for albumin concentration was calculated according to the following formula: corrected calcium (mmol/L) = measured calcium (mmol/L) + (0.025 × (40–[albumin (g/L)]). The reference values for plasma corrected calcium were 2.03 − 2.65 mmol/L (8.12 − 10.6 mg/dL) and for plasma phosphate were 0.74 − 1.52 mmol/L (2.29 − 4.71 mg/dL). Perioperative lipase data were not included in the study analysis because they were incomplete. Azathioprine was not used as an immunosuppressant in all patients at our center, and in addition, no steroid dose was included in the analysis because the two treatment groups at our center had the same steroid dose usage for patients in the perioperative period.

### Statistical analysis

The demographics, clinical characteristics, and laboratory results of patients were summarized with descriptive statistics. Continuous variables were reported as mean ± standard deviation for normally distributed variables or median with interquartile range for non-normally distributed variables. Categorical variables were expressed as number (*n*) and percentage (%). The Kolmogorov–Smirnov or Shapiro–Wilk tests were used to assess the normality of continuous variables. For comparisons of normally distributed continuous variables, Student’s *t*-test was used, while the Mann–Whitney *U*-test was applied for non-normally distributed variables. Categorical variables were compared using the chi-square or Fisher’s exact test. Potential risk factors associated with hyperamylasemia were evaluated through univariable logistic regression analysis. Statistically significant variables were included in multivariate logistic regression analysis and the corresponding odds ratio and 95% CI were calculated. The associations among the factors were assessed to prevent multicollinearity. Receiver operating characteristic (ROC) curves were used to assess the effectiveness of relevant metrics in predicting perioperative hyperamylasemia, and the optimal cutoff point was derived using the Youden index. All statistical analyses were performed using the SPSS version 29.0.0 (IBM SPSS Inc., Chicago, IL). A *p*-value ≤0.05 was considered statistically significant.

## Results

### Baseline characteristics and clinical parameters of the study population

A total of 584 KTRs who underwent kidney transplantation were included in our cohort from 2020 to 2023. Ultimately, 540 patients were included in the study. The screening process is shown in Figure 1.

All patient demographics and clinical data are detailed in Table 1, as well as the clinical and laboratory data that was collected on presentation. Among the KTRs, 153 (28.3%) had perioperative hyperamylasemia, 74 (13.71%) had hepatitis, 92 (17.04%) had chronic smoking, 60 (11.11%) had chronic drinking, 462 (85.56%) had hypertension, 88 (16.30%) had diabetes, 19 (3.52%) had brain infarction, 37 (6.85%) had CAD, 19 (3.52%) had PKD, 33 (6.11%) of the patients underwent preemptive kidney transplantation, 333 (61.67%) had preoperative serum amylase above normal values. We carefully reviewed the electronic medical record system, and in all patients included in the study, no history of prior pancreatitis-related illness was queried.

**Table 1.** Cohort baseline characteristics at presentation (*n* = 540).

Characteristic	Normal (*N* = 387)	Hyperamylasemia *N* = 153	*p*
Age (years)	43.95 ± 12.03	43.11 ± 12.08	0.467
Sex, female *n* (%)	121 (31.3)	55 (35.9)	0.296
BMI (kg/m^2^)	22.59 ± 3.79	22.25 ± 3.82	0.350
Past Medical History *n* (%)			
Hepatitis	58 (15)	16 (10.5)	0.168
Chronic smoking	71 (18.3)	21 (13.7)	0.198
Chronic drinking	43 (11.1)	17 (11.1)	1.000
Hypertension	334 (86.3)	128 (83.7)	0.431
Diabetes	66 (17.1)	22 (14.4)	0.448
Brain infarction	17 (4.4)	2 (1.3)	0.135
CAD	25 (6.5)	12 (7.8)	0.566
PKD	13 (3.4)	6 (3.9)	0.749
Dialysis type *n* (%)			0.310
non-dialysis	20 (5.2)	13 (8.5)	
peritoneal dialysis	86 (22.2)	30 (19.6)	
hemodialysis	281 (72.6)	110 (71.9)	
Immune induction regimen *n* (%)			0.050
ATG/ALG	191 (49.4)	70 (45.8)	
ATG/ALG + Basiliximab	165 (42.6)	60 (39.2)	
Basiliximab	31 (8)	23 (15)	
Laboratories on admission			
AST (U/L)	14.82 ± 6.51	16.02 ± 9.17	0.090
ALT (U/L)	15.60 ± 12.58	16.98 ± 12.40	0.249
Albumin (g/L)	42.52 ± 5.12	42.51 ± 5.03	0.980
TC (mmol/L)	4.27 ± 1.14	4.53 ± 1.30	0.022
TG (mmol/L)	1.88 ± 1.28	2.02 ± 1.52	0.298
LDL-C (mmol/L)	2.29 ± 0.88	2.56 ± 1.03	0.003
Serum calcium (mmol/L)	2.28 ± 0.21	2.28 ± 0.27	0.757
Serum phosphate (mmol/L)	1.87 ± 0.58	2.04 ± 0.61	0.002
Serum amylase (U/L)	132.34 ± 49.45	158.75 ± 49.67	<0.001
Type of CNIs n (%)			
TAC	351 (90.70)	134 (87.58)	0.996
High CNIs trough concentration *n* (%)	157 (40.6)	91 (59.5)	<0.001
DGF *n* (%)	120 (31)	55 (35.9)	0.214
LOS *d*	18.21 ± 11.07	19.27 ± 12.61	0.335

Abbreviations: BMI, body mass index; CAD, coronary heart disease; DGF, delayed graft function; PKD, polycystic kidney disease; ATG, anti-thymocyte globulin; ALG, antilymphocyte globulin; AST, aspartate aminotransferase; ALT, alanine aminotransferase; TAC, Tacrolimus; TC, total cholesterol; TG, triglycerides: LDL-C, low-density lipoprotein Cholesterol; CNIs, calcineurin inhibitors; LOS, Length of stay.

Three immunosuppressive drug regimens were used for induction at our center: anti-thymocyte globulin (ATG), anti-lymphocyte (ALG), and Basiliximab. Immune induction regimens were defined as multicategorical variables in the study, ATG/ALG, ATG/ALG+ basiliximab, and basiliximab, respectively. ATG/ALG was used as a reference in the analysis. A triple-drug immunosuppressive regimen, including a CNIs, an antimetabolic drug, and a steroid, was administered to all KTRs. CNIs include tacrolimus (TAC) and cyclosporine (CsA). Based on the clinical experience of our center, high CNIs trough concentration is defined as TAC trough concentration higher than 10 ng/ml or CsA concentration higher than 200 ng/ml, measured on the day of or 1 day before or after the highest postoperative serum amylase. Of all the data, there were 8 missing data for the high CNIs trough concentration, and these were filled in using SPSS multiple imputation. In addition there was no difference in the type of CNIs between the two groups of patients (*p* = 0.996).

### Factors affecting perioperative hyperamylasemia

The results of univariate analysis and multivariate logistic regression analysis in the non-hyperamylasemia cohort and hyperamylasemia cohort are shown in Table 2. The results of the univariate analysis showed that the third induction program: basiliximab (*p* = 0.022), TC (*p* = 0.024), LDL-C (*p* = 0.003), preoperative serum phosphate (*p* = 0.002), preoperative serum amylase (*p* < 0.001), and perioperative high CNIs trough concentration (*p*<.001) had a statistically significant effect on perioperative hyperamylasemia. TC and LDL-C measurements were normally distributed and were tested for Pearson correlation analysis, *r* = 0.822 (*p* < 0.001). Therefore, LDL-C was not included in the subsequent analysis. Finally, multifactorial logistic regression analysis showed that preoperative serum phosphate (*p* = 0.005), preoperative serum amylase (*p* < 0.001), and perioperative high CNIs trough concentration (*p* < 0.001) were identified as risk factors for perioperative hyperamylasemia. And the third induction program: basiliximab (*p* = 0.064), TC (*p* = 0.050) were not statistically significant.

**Table 2.** Univariate and multivariate logistic regression analysis of perioperative hyperamylasemia.

	Univariate logistic regression			Multiple logistic regression		
Predictors	*p*-value	OR	95% CI	*p*-value	OR	95% CI
Age	0.466	0.994	0.979-1.010			
Sex, female	0.296	1.234	0.833-1.830			
BMI	0.350	0.976	0.929-1.027			
Past Medical History						
Hepatitis	0.170	0.662	0.368-1.193			
Chronic smoking	0.200	0.708	0.418-1.200			
Chronic drinking	1.000	1.000	0.551-1.814			
Hypertension	0.431	0.812	0.484-1.363			
Diabetes	0.449	0.817	0.484-1.379			
Brain infarction	0.099	0.288	0.066-1.263			
CAD	0.567	1.232	0.603-2.520			
PKD	0.749	1.174	0.438-3.147			
Dialysis modalities						
Peritoneal dialysis	0.133	0.537	0.238-1.210			
Hemodialysis	0.175	0.602	0.290-1.252			
Induction programs						
ATG/ALG + Simulect	0.970	0.992	0.663-1.484	0.969	1.009	0.658 − 1.546
Basiliximab	0.022	2.024	1.106-3.707	0.064	1.845	0.965 − 3.527
Laboratories on admission						
AST	0.093	1.021	0.997-1.047			
ALT	0.263	1.008	0.994-1.023			
Albumin	0.980	1.000	0.963-1.037			
TC	0.024	1.194	1.023-1.394	0.050	1.845	1.000 − 1.393
TG	0.299	1.073	0.939-1.226			
LDL-C	0.003	1.348	1.106-1.642			
Serum calcium	0.756	0.877	0.384-2.004			
Serum phosphate	0.002	1.651	1.205-2.263	0.005	1.621	1.160 − 2.266
Serum amylase	0.001	1.010	1.007-1.014	0.001	1.01	1.006 − 1.015
Type of CNIs *n* (%)						
TAC	0.996	1.002	0.513-1.956			
High CNIs trough concentration	0.001	2.130	1.448-3.133	0.001	2.335	1.560 − 3.494
DGF	0.214	1.286	0.865-1.914			

Abbreviations: CI, confidence interval; BMI, body mass index; CAD, coronary heart disease; DGF, delayed graft function; PKD, polycystic kidney disease; ATG, anti-thymocyte globulin; ALG, antilymphocyte globulin; AST, aspartate aminotransferase; ALT, alanine aminotransferase; TAC, Tacrolimus; TC, total cholesterol; TG, triglycerides: LDL-C, low-density lipoprotein Cholesterol; CNIs, calcineurin inhibitors.

In the hyperamylasemia group, a total of 11 patients were eventually diagnosed with AP, one died of sepsis from moderately severe AP, and the rest had acute mild pancreatitis and all survived. Some of the above patients had missing follow-up, so whether the patients had post-transplantation diabetes mellitus or chronic pancreatitis was not analyzed.

### Predictive value of perioperative hyperamylasemia

**Figure 2.**
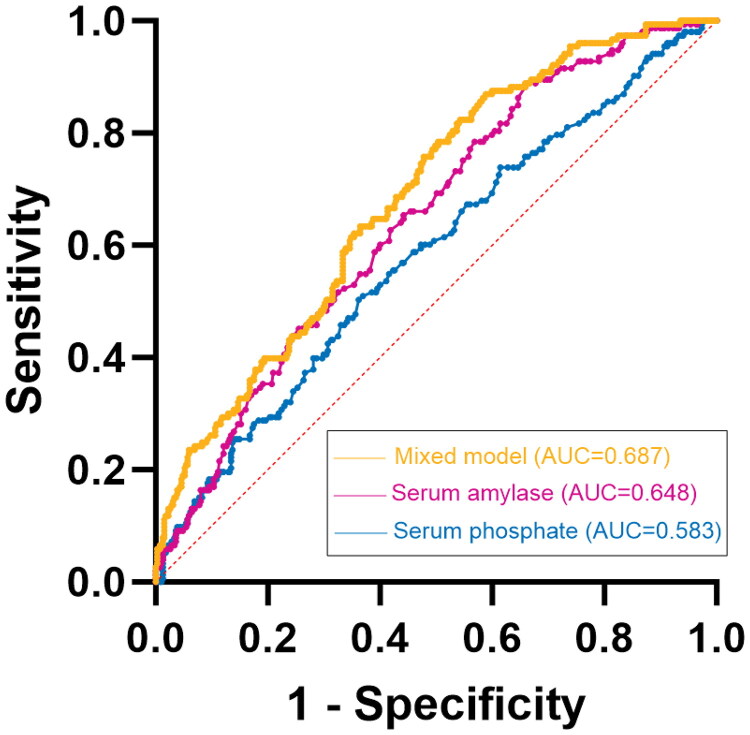
ROC Curves for relevant indicators.

ROC curve analysis was used to assess the predictive value of the above metrics for perioperative hyperamylasemia (Figure 2), and the Youden index, sensitivity, specificity, cutoff point, the area under the ROC curve (AUC) and 95% CI were listed in Table 3. The AUC of the serum phosphate was 0.583 (95% CI: 0.529-0.637); and the AUC of the Serum amylase was 0.648 (95% CI: 0.599–0.697). Given that high CNIs concentration is a dichotomous variable, its predictive value was not analyzed using ROC curve analysis. The sensitivity and specificity for diagnosing perioperative hyperamylasemia are 0.595 and 0.594, respectively. Finally, we used serum amylase, serum phosphate and high CNIs concentration as a mixed model to predict perioperative hyperamylasemia, and the ROC was 0.687 (0.64–0.734). It can be seen that the ROC value is further improved.

**Table 3.** Predictive value of relevant indicators for perioperative hyperamylasemia.

indicators	Cutoff value	Sensitivity	Specificity	Youden index	AUC (95% CI)
Serum phosphate	1.995	0.503	0.638	0.142	0.583(0.529–0.637)
Serum amylase	108.500	0.889	0.336	0.225	0.648(0.599–0.697)
High CNIs concentration		0.595	0.594		
Mixed Model	0.202	0.869	0.413	0.283	0.687(0.64–0.734)

Abbreviations: AUC, the area under the ROC curve; CI, confidence interval. Mixed model: a combination of serum phosphate, serum amylase, and high CNIs concentrations.

## Discussion

The retrospective study examined the factors influencing perioperative hyperamylasemia in KTRs. Our study demonstrates that elevated preoperative serum phosphate, preoperative serum amylase, and perioperative high CNIs trough concentration are risk factors for perioperative hyperamylasemia.

Decreased metabolic clearance of amylase, indicated by decreased renal filtration and reabsorption, leads to increased serum amylase level [7]. All renal transplant recipients suffered from renal insufficiency. Vaziri et al. [9] reported that serum amylase and lipase levels are significantly elevated in patients with end-stage renal disease (ESRD) in the absence of clinical pancreatitis and the hyperamylasemia was not associated with increased P3 isoamylase levels unless pancreatitis was present. Our study observed similar results. In our study, 333 (61.67%) of patients had preoperative serum amylase levels above normal values (139.82 ± 50.88). Collen et al. [10] examined serum amylase values in 128 subjects with creatinine clearances below 90 mL/min. They found that serum amylase remained normal when creatinine clearance was above 50 mL/min, and did not elevate until creatinine clearance was below 50 mL/min. This illustrates the close correlation between preoperative amylase levels and renal function in KTRs. Our study found that preoperative serum amylase levels are a risk factor for perioperative hyperamylasemia. We believe the potential mechanisms are as follows: During the perioperative period, conditions such as increased stress, trauma, fluid imbalance, infection, and inflammation, enhance the body’s metabolism and amylase secretion. Before graft filtration function is restored, a lower glomerular filtration rate (GFR) leads to higher serum amylase levels, increasing hyperamylasemia incidence. Liu et al. [11] investigated the incidence and identified risk factors for postoperative hyperamylasemia and pancreatitis following total knee arthroplasty. They found that a significant percentage (25.3%) of patients developed hyperamylasemia after total knee arthroplasty. Patients with higher hypertriglyceridemia, higher preoperative serum amylase, and intra-operative blood loss had an increased risk for postoperative hyperamylasemia and pancreatitis. Our findings are partly consistent with theirs.

Furthermore, an extensive and systematic search for perioperative hyperamylasemia and postoperative hyperamylasemia on PubMed and Embase. The International Study Group of Pancreatic Surgery defines postoperative serum hyperamylasemia as serum amylase exceeding the institution’s upper limit of normal within 48 h postoperatively [12]. However, this definition is intended for diagnosing postpancreatectomy pancreatitis. Kidney transplant surgeries do not affect the pancreas. Therefore, this definition is not applicable to our study. Additionally, we found no reports of perioperative hyperamylasemia in KTRs. Perioperative hyperamylasemia can occur during abdominal and non-abdominal surgeries [11,13–15]. Morrissey et al. [15] studied isoenzyme patterns of the serum amylase elevation in patients undergoing various surgical procedures. When amylase levels are significantly elevated postoperatively, the peak usually occurs about 24 h after surgery. Hyperamylasemia within 48 h after surgery may be associated with surgical procedures, including metabolic responses to trauma, the use of anesthetics (morphine and other analgesics), and nonspecific responses mediated by stress-provoked mechanisms. In our study, 255 patients (47.22%) experienced hyperamylasemia 48 h after surgery. We hypothesize that this is associated with pancreatic injury.

Inorganic phosphate is essential for intracellular signal transduction, energy metabolism, cell membrane formation, DNA and RNA synthesis, and bone mineralization [16]. Deranged calcium-phosphorus metabolism frequently occurs in chronic kidney disease (CKD) [17], progressively worsens as patients approach ESRD [18], and is not fully reversed after kidney transplantation [19–21]. Among patients with ESRD, loss of renal function is often accompanied by hyperphosphatemia, deficiencies of 25-hydroxyvitamin D3 and/or 1,25-hydroxyvitamin D3, and increased levels of the phosphatonin fibroblast growth factor23 [21–23]. These markers of disturbed calcium and phosphorus metabolism are causal pathways linking "phosphorus toxicity" and its clinical consequences [24,25]. Although further research is needed, dysregulation of phosphate homeostasis may impair insulin signaling, thereby triggering insulin resistance and decreasing cellular utilization of glucose. Nguyen et al. [26] reported that elevated extracellular inorganic phosphate causes mitochondrial oxidative stress linked to mitochondrial hyperpolarization. Such stress results in reduced insulin content defective insulin secretion and cytotoxicity. This indicates that high phosphate levels cause some degree of pancreatic damage. Our study found that preoperative serum phosphate is a risk factor for perioperative hyperamylasemia. This may result from pancreatic damage caused by elevated preoperative phosphate levels.

Many clinical studies have shown a strong association between hyperphosphatemia and the severity of AP [27–29]. Mazzini et al. [30] reported that serum phosphate correlates with severity in AP and implicates extracellular purines in the systemic response to severe AP. They found that severe AP led to increased serum levels of adenosine diphosphate, adenosine monophosphate, and adenosine, with a positive correlation between serum adenosine and phosphate in the AP groups. In conclusion, hyperphosphatemia correlates with pancreatitis severity, and some studies suggest that it may lead to pancreatic injury. However, relatively few studies have explored this connection, indicating a need for more basic research.

Our research also found that perioperative high CNIs trough concentrations are a risk factor for perioperative hyperamylasemia. Our center observed that during perioperative periods, patients experienced adverse reactions, such as hand tremors, when serum TAC trough concentrations exceeded 10 ng/ml or CsA trough concentrations exceeded 200 ng/ml. Given that our recipients were all Asian, the drug dose was less well-tolerated compared to Europeans and Americans. Drug-induced AP is a rare cause of AP, accounting approximately 0.1–5% of all AP cases [31]. AP has been linked to immunosuppressive agents, with azathioprine being the most commonly implicated drug, followed by steroids, as supported by substantial evidence [32]. Recent case reports have highlighted TAC and CsA as potential causes of AP [33–37]. Maruyama et al. [35] reported a case of AP 67 days after kidney transplantation. In this case, an extremely high trough concentration of TAC (>30 ng/ml) coincided with the onset of pancreatitis. Yang et al. [38] used the Food and Drug Administration Adverse Event Reporting System to investigate the risk signal of AP associated with CNIs. They identified 221 cases of AP linked to CNIs. This study demonstrated a significant association between CNIs and AP (ROR 1.82 [1.60 − 2.08], IC 0.85 [3.66 − 3.92]). CNIs have diabetogenic potential and can cause pancreatic islet cell damage, decreased insulin secretion, and peripheral insulin resistance [39]. Animals treated with TAC have shown significantly more acinar cell necrosis [40]. Accumulation of CNIs in the pancreas may lead to TAC-induced pancreatitis. The mechanisms behind CNIs-induced AP may involve immunologic reactions, cell metabolism, and systemic or local infections. some studies propose that TAC causes a decline in the activity and plasma concentration of lipoprotein lipase, leading to hypertriglyceridemia, which is an independent risk factor for AP [41,42]. Chen et al. [43] investigated the mechanism of TAC-induced pancreatic injury and to explore the potential effect from small dose of sirolimus. Wistar rats were randomly divided normal control group, post transplantation diabetes mellitus group, sirolimus intervention group. They found that transcriptomic analysis indicated Syk/BLNK/NF-κB signaling was significantly up-regulated in post transplantation diabetes mellitus group, and pathological staining, immumohistochemical staining, immunofluorescent staining, western blot verified Syk/BLNK/NF-κB and TNF-α/IL-1β were all significantly increased (*p* < 0.05 or *p* < 0.01), demonstrating the mechanism of TAC-induced pancreatic injury *via* Syk/BLNK/NF-κB signaling. Triñanes et al. [44] evaluated the effect of TAC, CsA, and metabolic stressors (glucose plus palmitate) on insulinoma β cells *in vitro* and in pancreata of obese and lean Zucker rats. TAC potentiates glucolipotoxicity in β cells, possibly by sharing common pathways of β cell dysfunction. TAC-induced β cell dysfunction is potentially reversible. Inhibition of the calcineurin-NFAT pathway may contribute to the diabetogenic effect of CNIs but does not explain the stronger effect of TAC compared with CsA. Several studies have shown that Tac is toxic to β-cells in the pancreas and that oxidative damage plays a central role in Tac-induced β-cell dysfunction [45,46]. Drachenberg et al. [47] studied 26 pancreas allograft biopsies, performed 1-8 months post-transplantation, from 20 simultaneous kidney-pancreas transplant recipients, randomized to receive either TAC or CsA. On light microscopy cytoplasmic swelling, vacuolization, apoptosis, and abnormal immunostaining for insulin were seen in biopsies from patients receiving either TAC or CsA. The islet cell damage was more frequent and severe in the group receiving TAC than in the group receiving CsA (10/13 and 5/13, respectively) but the differences were not statistically significant. Significant correlation was seen between the presence of islet cell damage and serum levels of CsA or TAC during the 15 days previous to the biopsy, as well as with the peak level of TAC. Toxic levels of CsA or TAC and administration of pulse steroids were associated with hyperglycemia when these occurred concurrently (*p* = 0.005). This study found impairment of pancreatic beta cell damage by CNI. In addition toxic levels of CsA or TAC and higher steroid doses enhance each other’s diabetogenic effects. In conclusion, the above studies revealed some mechanisms of pancreatic injury by CNI drugs. In conclusion, the above studies reveal the toxic effects of CNI on the pancreas. This explains, to some extent, why high concentrations of CNI lead to hyperamylasemia in KTRs.

In addition, our study also found that the difference in the incidence of DGF was not statistically significant between the normal and hyperamylasemia groups of patients, which were 31 and 35.9%, respectively, *p* = 0.214. Comai et al. found that patients with concomitant amylase increase > 20% and RI > 0.7 might require closer monitoring to diagnose DGF early and modify the therapeutic approach accordingly [48]. Their study measured before and after transplantation, and at discharge. The amylase measurements in this study were taken before and after transplantation and at discharge. In contrast, the patients with hyperamylasemia in our study were treated symptomatically as soon as it was detected, and most of the amylases decreased to normal when discharged. In addition, our study had a larger sample size. The above reasons may lead to different results from our study.

Several limitations are noted in this study. First, this study is a retrospective review of patients at a single institution, where re-interventions or amputation may be underestimated due to uncaptured events. Second, accurate assessment of pancreatic injury was hindered by incomplete data on perioperative lipase measurements. Third, due to cost constraints, amylase isoform testing was not performed, preventing accurate differentiation between pancreatic and salivary sources of hyperamylasemia during the perioperative period. Fourthly, perioperative cytomegalovirus (CMV) reactivation may theoretically affect patient amylase levels. However, preoperative cytomegalovirus levels were not tested in all patients at our center, and were usually tested periodically starting 2 weeks after surgery. Therefore, reactivation of CMV was not included in the study, and subsequent testing for viral levels of CMV could be refined to further assess its correlation with hyperamylasemia. Fifthly, the CNIs in our center are predominantly TAC, with a smaller number of patient cases with CsA. Although there was no statistical difference between the effects of TAC and CsA on hyperamylasemia in this study. However, limited by the sample size of CsA, its statistical efficacy may not be convincing. The sample size of CsA should be enlarged in the follow-up, so that the effects of different CNIs on hyperamylasemia can be more accurately assessed. Thus, prospective studies are warranted to explore the causes and sources of perioperative hyperamylasemia.

To prevent perioperative hyperamylasemia in KTRs, we recommend that clinicians tailor appropriate prevention strategies based on clinical needs and risk factors, including controlling preoperative phosphate levels, monitoring CNI levels, and optimizing perioperative management. First, patients undergoing proposed kidney transplantation should be adequately educated and advised to be adequately dialyzed to improve preoperative phosphate and amylase levels. Second, we believe that the following patients belong to the high-risk group of perioperative hyperamylasemia: patients with preoperative hyperamylasemia, hyperphosphatemia, and patients who develop high CNIs concentrations in the perioperative period. It is recommended to be more cautious in the face of the above high-risk groups, to closely monitor their perioperative serum amylase changes, and to actively intervene pharmacologically, if necessary, to control hyperamylasemia, so as to optimize the perioperative management of high-risk groups. We must pay attention to perioperative hyperamylasemia in KTRs, which is a precursor to pancreatitis or pancreatic injury. Reasonable preventive strategies can reduce the incidence of perioperative hyperamylasemia, shorten the length of patient hospitalization, and reduce medical expenditures.

In conclusion, elevated preoperative serum phosphate levels, preoperative serum amylase levels, and high perioperative CNIs trough concentrations are risk factors for perioperative hyperamylasemia. This study may provide valuable insights for clinicians to identify the causes of perioperative hyperamylasemia and formulate prevention and treatment strategies. However, large sample sizes and multi­center studies are still needed to verify this conclusion, and the mechanism requires further research.

## Data Availability

The raw data supporting the conclusions of this article will be made available by the authors, without undue reservation.
